# Acute *Coxiella burnetii* Infection: A 10-Year Clinical Experience at a Tertiary Care Center in the United States

**DOI:** 10.1093/ofid/ofae277

**Published:** 2024-05-10

**Authors:** Said El Zein, Doug W Challener, Nischal Ranganath, Ryan B Khodadadi, Elitza S Theel, Omar M Abu Saleh

**Affiliations:** Division of Public Health, Infectious Diseases and Occupational Medicine, Department of Medicine, Mayo Clinic, Rochester, Minnesota, USA; Division of Public Health, Infectious Diseases and Occupational Medicine, Department of Medicine, Mayo Clinic, Rochester, Minnesota, USA; Division of Public Health, Infectious Diseases and Occupational Medicine, Department of Medicine, Mayo Clinic, Rochester, Minnesota, USA; Division of Public Health, Infectious Diseases and Occupational Medicine, Department of Medicine, Mayo Clinic, Rochester, Minnesota, USA; Division of Clinical Microbiology, Department of Laboratory Medicine and Pathology, Mayo Clinic, Rochester, Minnesota, USA; Division of Public Health, Infectious Diseases and Occupational Medicine, Department of Medicine, Mayo Clinic, Rochester, Minnesota, USA

**Keywords:** acute Q fever, *Coxiella burnetii*, diagnosis, treatment, anticardiolipin, hydroxychloroquine

## Abstract

**Background:**

Identifying and treating patients with acute Q fever who are at an increased risk of progressing to persistent disease is crucial for preventing future complications. In this study, we share our decade-long clinical experience with acute Q fever, highlighting the challenges that clinicians encounter from making an initial diagnosis and performing risk stratification to determining the appropriate prophylaxis regimen and duration.

**Methods:**

We retrieved records of adult Mayo Clinic patients (≥18 years) with positive *Coxiella burnetii* serology results between 1 January 2012 and 31 March 2022. Patients with Q fever anti–phase II immunoglobulin G ≥1:256 by indirect immunofluorescence were further analyzed.

**Results:**

Thirty-one patients were included. Their median age was 58 years (IQR, 50–64), and the majority were men (84%). Acute hepatitis (29%), flu-like illness (25.8%), and pneumonia (16%) were the most common presentations. Thirteen patients (42%) received antibiotic prophylaxis to prevent disease progression, with significant variation in the indications and duration across physicians. The combination of doxycycline and hydroxychloroquine was the preferred regimen. Prophylaxis was administered for a median 333 days (IQR, 168–414). Four patients (13%) progressed to Q fever native valve infective endocarditis, with elevated anticardiolipin immunoglobulin G levels being the sole risk factor in 2 cases. The small sample size precluded drawing conclusions on the impact of prophylaxis in preventing disease progression.

**Conclusions:**

Management of acute Q fever is complicated by the lack of comprehensive clinical guidelines leading to varied clinical practices. There is a critical need for randomized trials to establish robust evidence-based protocols for management.

## Teaser

Acute Q fever presents diagnostic challenges owing to nonspecific clinical presentations. Management complexities arise from the lack of consensus on “high risk” factors for disease progression and the absence of streamlined treatment protocols. Randomized trials are needed to help establish evidence-based guidelines.

Acute Q fever, a zoonotic disease caused by *Coxiella burnetii*, poses significant diagnostic challenges for physicians. Symptoms are present in half of individuals acutely infected [1, 2] and are often nonspecific and self-limited. Progression to focal persistent disease occurs in <5% of cases following the primary acute infection [3, 4]. The 10-year average annual incidence of acute Q fever in the United States is an estimated 0.36 cases per million persons, almost doubling the annual incidence from 0.3 cases in 2008 to 0.5 cases in 2017 [5]. While acute Q fever diagnosis remains rare overall, infections appear to be more prevalent in the West North Central states [5, 6], representing the “Agricultural Heartland” of the United States, likely due to a higher probability of contact with animals and animal products [3, 6]. According to the Centers for Disease Control and Prevention (CDC), the highest incidence of Q fever infections in 2019 was reported in South Dakota (12.43 cases per million persons), followed by Iowa (5.71 cases) [7].

Despite its typically self-limiting nature, accurately identifying patients at increased risk of progressing to focal persistent disease is essential for establishing close clinical follow-up, guiding therapeutic strategies, and preventing future complications. However, this is complicated by the ambiguities surrounding the choice of diagnostic tests for patient risk stratification and the determination of specific host conditions that constitute a high risk for disease progression. The 2013 CDC guidelines [3] provide a framework for clinicians in the United States but fall short in offering detailed recommendations for risk factor assessment, leaving much to the clinical judgment of health care providers. Furthermore, the guidelines lack specific recommendations regarding the choice and duration of treatment for patients at high risk. This absence of high-quality data derived from randomized clinical trials can lead to variability in clinical practice and may affect patient outcomes.

In this study, we share our decade-long clinical experience with acute Q fever, highlighting the myriad challenges that clinicians face, from making an initial diagnosis and performing risk stratification to determining the appropriate prophylaxis regimen and duration.

## METHODS

### Case Definition

We retrieved records of all Mayo Clinic patients seen at the Minnesota, Arizona, and Florida campuses with a positive *C burnetii* serologic test result reported between 1 January 2012 and 31 March 2022.

Patients aged ≥18 years who had a Q fever anti–phase II immunoglobulin G (IgG) ≥1:256 by indirect immunofluorescence were further analyzed [8]. Patients with an anti–phase II IgG <1:256 were included only if they had a 4-fold rise in titers at follow-up within 3 to 6 weeks. The 2009 case definition and classification developed by the Council of State and Territorial Epidemiologists and adopted by the CDC were utilized to categorize patients into confirmed and probable cases of acute Q fever, as well as confirmed cases of focal persistent Q fever [3, 9]. Patients without clinical evidence of infection were excluded.

Throughout this study, “focal persistent disease” describes patients with an identifiable focus of *C burnetii* organ involvement (eg, osteoarticular, endovascular, or endocardial infection) in the setting of elevated phase I IgG titers (≥1:1024) and/or other consistent laboratory criteria [3, 10, 11]. The traditional serologic definition of “chronic Q fever” describes the serologic pattern of phase I IgG ≥1:1024 irrespective of evidence of a persistent focus.

### Laboratory Techniques

All patients were tested by the *C burnetii* immunofluorescence assay (Focus Diagnostics) to determine immunoglobulin M (IgM) and IgG antibody titers against phase I and II antigens. Briefly, patient serum undergoes 2-fold serial dilution starting at 1:16 and is overlayed onto inactivated *C burnetii* cells immobilized on a glass slide. Following incubation, slides are washed and incubated with fluorescein-labeled anti-human IgG or IgM conjugate. Following a final wash step, slides are examined for fluorescence. The highest serum dilution that reveals definite fluorescence is reported as the end point titer.

Molecular detection of *C burnetii* in tissue and serum was performed by real-time polymerase chain reaction (PCR), targeting the unique shikimate dehydrogenase gene (aroE) sequence specific to *C burnetii*, as previously described [12, 13].

Anticardiolipin (aCL) IgG antibodies (QUANTA Lite ACA IgGII; Inova Diagnostics) in serum were measured as previously described and were considered positive at ≥15 IgG phospholipid units (GPL) [14].

### Study Definitions

In the absence of a universally accepted consensus on what constitutes “high risk” for disease progression, we used criteria that align with the current medical literature (Table 1).

**Table 1.** Common Risk Factors for Progression to Focal Persistent Disease Reported in the Literature

Author	Year	Country	No. of Patients	Risk Factor for Progression	Persistent Focalized Q Fever Syndrome
Melenotte [11]	2018	France	2434	Lymphadenitis, hemophagocytic syndrome	Lymphoma
				Valvulopathy, thrombosis, lymphadenitis, phase I IgG titer >800, acute Q fever endocarditis	Persistent focal infection
				Elevated anticardiolipin IgG^a^	Infective endocarditis Lymphoma
Million [15]	2014	France	4	Joint prosthesis	Prosthetic joint septic arthritis
Million [16]	2013	France	72	Age ≥40 y,^b^ valvulopathy	Infective endocarditis
Million [17]	2013	France	72	Age >40 y, elevated anticardiolipin IgG, elevated phase II IgM	Infective endocarditis
Kampschreur [4]	2012	Netherlands	105	Valvular surgery, vascular prosthesis, aneurysms, renal insufficiency,^c^ age >60 y	Endovascular infection Infective endocarditis
Carcopino [18]	2007	France	54	Pregnancy	“Chronic Q fever,” especially infective endocarditis
Fenollar [19]	2001	France	1569	Valvulopathy including valve prosthesis	Infective endocarditis
Stein [20]	1998	France	5	Pregnancy	“Chronic infection”
Raould [21]	1992	France	5	Immunosuppression	Infective endocarditis
Heard [22]	1985	UK	5	Immunosuppression	“Chronic Q fever”

This table does not represent an exhaustive list of studies. Its purpose is to highlight various factors deemed “high risk” for disease progression and the rationale for choosing specific risk factors in our study.

Abbreviations: IgG, immunoglobulin G; IgM, immunoglobulin M.

^a^Elevated anticardiolipin IgG is associated with several complications: acute Q fever endocarditis, hemophagocytic syndrome, hepatitis, cholecystitis, thrombosis, meningitis, and persistent endocarditis. Acute Q fever endocarditis itself is a risk factor for persistent endocarditis. Hemophagocytic syndrome is a risk factor for lymphoma.

^b^Older patients are more likely to have valvulopathies.

^c^Renal insufficiency is associated with vasculopathy.

These included the presence of valvopathy, vascular grafts, vascular aneurysms, pregnancy, elevated aCL IgG antibody level, and/or immunosuppression. For the purposes of this study, patients older than 40 years who lacked other high-risk factors were not considered to be at high risk for disease progression.

Other study definitions are outlined in the supplementary appendix.

### Data Collection and Statistical Analysis

Data were collected pertaining to patient demographics, medical comorbidities, clinical presentation, laboratory evaluations, radiographic imaging, treatment, and outcomes. Patient records were reviewed up to the date of their last evaluation by the infectious diseases team to document their outcomes. Patient characteristics were summarized with frequency (percentage) for categorical variables and median (IQR) for continuous variables. All analyses were conducted with R version 4.2.2 (R Foundation for Statistical Computing).

## RESULTS

### Patient Demographics and Clinical Presentation

**Figure 1.**
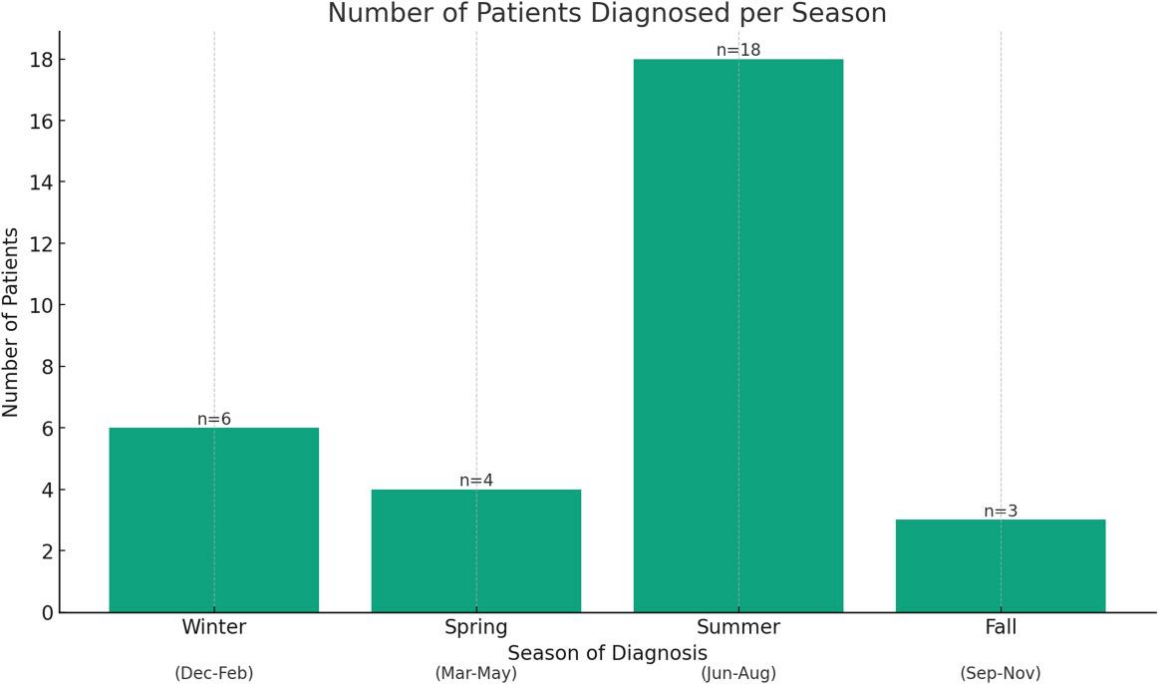
Seasonal distribution of acute Q fever diagnoses.

A total of 31 patients were included in this study, the majority residing in the Midwest (n = 19, 61%; Table 2). Most patients (n = 18, 58%) were diagnosed in the summer (1 June–31 August; Figure 1). According to the CDC case classification, 22 patients (71%) had probable acute Q fever while 9 (29%) had confirmed acute Q fever (Supplementary Figure 1, Supplementary Table 1). On average, there appears to be a slight increase in the number of cases diagnosed over the past 5 years as compared with previous years (Supplementary Figure 2). The median age was 58 years (IQR, 50–64) and the majority were men (n = 26, 84%). Six patients (19%) had no identifiable exposure history. Acute hepatitis (n = 9, 29%), isolated flu-like illness (n = 8, 25.8%), and pneumonia (n = 5, 16%) were the most common clinical presentations. Notably, flu-like symptoms were reported in the majority of patients (n = 28, 90.3%). Other rare presenting syndromes attributed to acute Q fever in the absence of identifiable alternative etiologies included meningoencephalitis (n = 1), membranoproliferative glomerulonephritis (n = 1), hemophagocytic lymphohistiocytosis (n = 1), acute lymphadenitis (n = 2), interstitial edematous pancreatitis (n = 1), and acute Q fever endocarditis (n = 1).

**Table 2.** Baseline Characteristics (N = 31)

	Median (IQR) or No. (%)
Age, y	58.0 (50.0–64.0)
Gender: women	5 (16.1)
Pregnant	0 (0)
State of residence	
Minnesota	12 (38.7)
Wisconsin	4 (12.9)
Iowa	2 (6.5)
Michigan	1 (3.2)
Arizona	5 (16.1)
Other^a^	7 (22.6)
Exposure history	
Contact with farm animals	27 (87.1)
Living in close proximity to farms	4 (12.9)
Abattoir worker	1 (3.2)
Unpasteurized milk intake	1 (3.2)
No known exposure	6 (19.3)
Comorbidities	
Diabetes mellitus	4 (12.9)
Chronic kidney disease	4 (12.9)
Solid malignancy	3 (9.7)
Hematologic malignancy	2 (6.5)
Autoimmune disease	3 (9.7)
Other^b^	4 (12.9)
Clinical presentation	
Flu-like symptoms	28 (90.3)
Isolated febrile illness	8 (25.8)
Acute hepatitis	9 (29.0)
Pneumonia	5 (16.1)
Acute lymphadenitis	2 (6.5)
Other^c^	5 (16.1)
Duration of symptoms prior to presentation, d	4.0 (2.0–7.5)

^a^California (n = 2), Georgia (n = 1), Montana (n = 1), Nevada (n = 1), Washington (n = 1), United Arab Emirates (n = 1).

^b^Solid organ transplant recipient (n = 1), history of prosthetic valve replacement (n = 1), presence of vascular graft (n = 1), aneurysm (n = 1), bicuspid aortic valve with mild stenosis (n = 1), asplenia (n = 1).

^c^Meningoencephalitis (n = 1), membranoproliferative glomerulonephritis (n = 1), hemophagocytic lymphohistiocytosis (n = 1), interstitial edematous pancreatitis (n = 1), acute Q fever endocarditis (n = 1).

### Laboratory and Imaging Findings

At the time of initial evaluation, the median phase II IgG and IgM titers were 1:2048 (range, 1:16–1:32 768) and 1:512 (range, 1:16–1:3200), respectively (Table 3). Four patients had concomitant elevated phase I IgG (≥1:1024) despite the absence of clinical and radiologic evidence of focal persistent disease (supplementary appendix). Serum PCR testing for *C burnetii* was obtained in 17 patients at the time of presentation to clinical care, all of which returned negative. Plasma microbial cell-free DNA next-generation sequencing (Karius Test; Karius Inc) was obtained in 5 patients at the time of presentation, out of which 3 returned positive for *C burnetii* despite a negative result from serum *C burnetii* PCR. aCL IgG titers were measured in only 8 patients on initial presentation (26%), with 6 (75%) showing elevated levels. The median aCL IgG titer was 96.1 GPL (IQR, 68.0–125.3; reference range, ≤15 GPL). Concomitant positive serology results for *Bartonella* spp, *Anaplasma phagocytophilum*, *Ehrlichia* spp, and *Borrelia burgdorferi* were noted in few cases (Supplementary Table 2).

**Table 3.** Laboratory and Imaging Results

	Median (IQR) or No. (%)
Laboratory workup on initial presentation	
IgG titer^a^	
Phase I	1:16 (1:16–1:8192)
Phase II	1:2048 (1:16–1:32 768)
IgM titer^a^	
Phase I	1:16 (1:16–1:1024)
Phase II	1:512 (1:16–1:3200)
*Coxiella burnetii* PCR positive	18 (62.1)
Serum	0/17 (0)
Tissue sample	1/1 (100)
Neutropenia, <1.5 ×10^9^/L	0 (0)
Thrombocytopenia, <135 ×10^9^/L	4 (12.9)
Anemia, <12.5 g/dL	11 (36.7)
ESR	
Elevated, >22 mm/1 h	13/20 (65)
Level, mm/1 h	76.0 (44.0–100.0)
CRP	
Elevated, >8.0 mg/L	16/26 (61.5)
Level, mg/L	98.0 (64.6–188.5)
aCL IgG antibodies	8 (25.8)
Elevated^b^	6 (75)
Level, GPL	96.1 (68.0–125.3)
Imaging	
Cardiac imaging obtained on initial presentation	25 (80.6)
TTE	17 (54.8)
Time between presentation and TTE, d	4.0 (2.0–11.0)
Abnormal TTE findings	5 (29.4)
TEE	11 (35.5)
Time between presentation and TEE, d	5.0 (1.0–12.0)
Abnormal TEE findings	6 (54.5)
PET-CT scan	10 (32.3)
Time between presentation and PET-CT, d	5.5 (3.2–7.8)
Abnormal PET-CT findings	6 (60.0)

Abbreviations: aCL, anticardiolipin; CRP, C-reactive protein; ESR, erythrocyte sedimentation rate; GPL, IgG phospholipid unit; IgG, immunoglobulin G; IgM, immunoglobulin M; PCR, polymerase chain reaction; PET-CT, positron emission tomography–computed tomography; TEE, transesophageal echocardiogram; TTE, transthoracic echocardiogram.

^a^Median (range).

^b^Anticardiolipin IgG elevated (>15 GPL; 1 GPL = 1 μg of IgG antibody).

Overall, 25 patients (81%) underwent echocardiography at the time of initial presentation, including 17 transthoracic echocardiograms (TTEs) and 8 transesophageal echocardiograms (TEEs). Following initial TTE, TEE was performed in 3 patients for further characterization of valvular abnormalities (Table 3). Abnormal valvular findings posing a high risk for progression to Q fever endocarditis were noted in 5 of 17 (29%) TTEs and 6 of 11 (54%) TEEs as detailed in Supplementary Table 3.

Ten patients (32%) had a PET-CT scan (positron emission tomography–computed tomography) performed on initial presentation (median, 5.5 days following admission; Supplementary Table 3), out of which only 1 had phase I IgG >1:1024. The PET-CT results changed management in 1 patient only: following identification of deep 18F-fluorodeoxyglucose–avid lymphadenopathy above and below the diaphragm associated with splenic involvement, a lymph node biopsy was performed, which did not show evidence of lymphoma.

### Clinical Management and Outcomes

The treatment regimen, criteria for antibiotic prophylaxis, and total duration of therapy for acute Q fever varied widely in our cohort. Doxycycline monotherapy was used in 21 patients (70%) at the time of initial diagnosis (Table 4). For those not requiring extended prophylaxis, the median duration of doxycycline administration was 14 days (IQR, 14–21).

**Table 4.** Treatment and Outcome

	Median (IQR) or No. (%)
Antibiotics administered	30 (96.8)
Doxycycline	21 (70)
Combination of doxycycline and hydroxychloroquine	8 (26.7)
Trimethoprim-sulfamethoxazole	1 (3.3)
Duration of antibiotic therapy received following initial diagnosis, d	22.0 (14.0–186.0)
Risk factors for progression to focal persistent disease	14 (45.2)
Patients received prolonged antibiotic prophylaxis	13 (42.0)
Duration of prophylaxis, d	333.0 (168.0–414.0)
Outcome of the acute infection	
Clinical resolution	22 (70.9)
Serologic progression only (phase I IgG ≥1:1024 during follow-up)^a^	7 (31.8)
Time between initial diagnosis and serologic progression, d	179 (63–358)
Progression to focal persistent disease	4 (12.9)
Native valve infective endocarditis	4 (100)
Time to diagnosis of native valve endocarditis, mo	4.5 (3.5–5.5)
Loss to follow-up	5 (16.1)

Abbreviation: IgG, immunoglobulin G.

^a^Defined as a patient with acute Q fever who, at any time during the evaluation, had a detectable phase I IgG titer ≥1:1024 but without clinical evidence of progression to focal persistent disease on subsequent follow-up.

**Figure 2.**
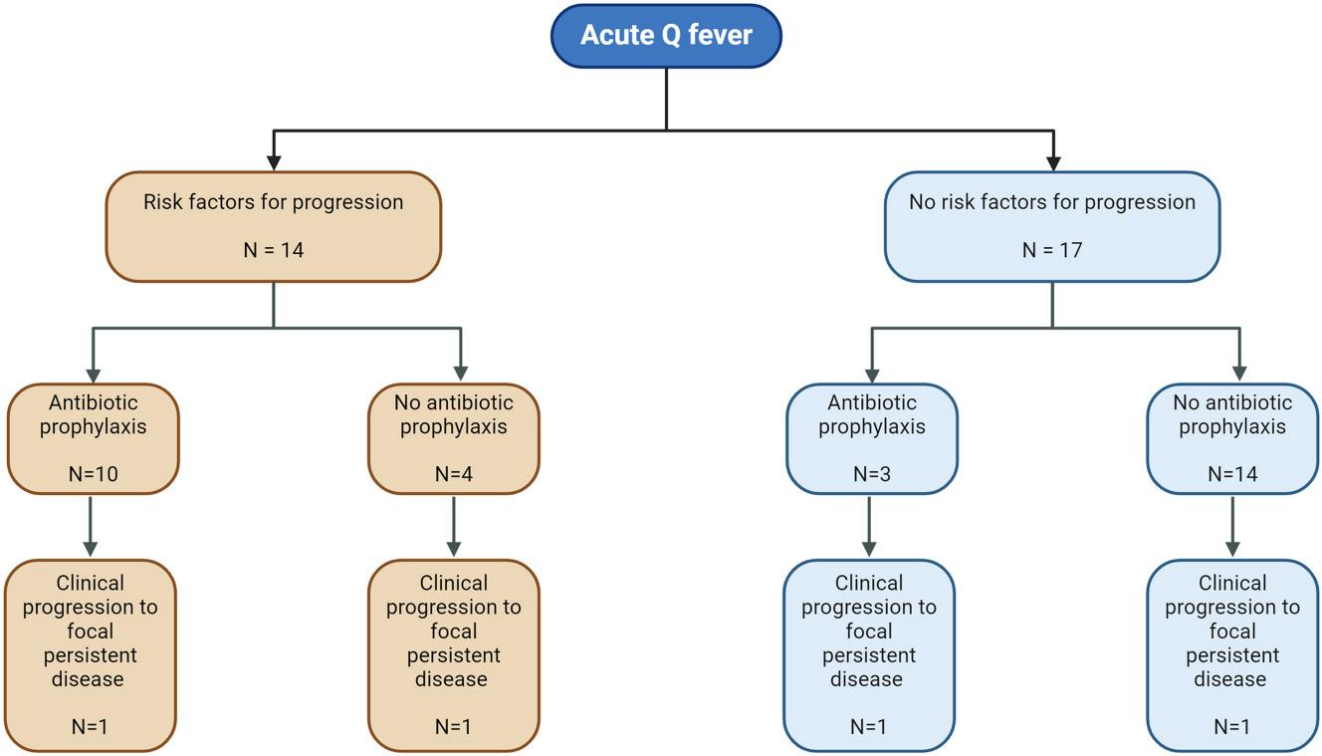
Treatment outcomes and disease progression in patients with acute Q fever based on risk factors and antibiotic prophylaxis.

None of the women in the cohort were pregnant. Fourteen patients were identified as being at high risk of progression to focal persistent Q fever per the study criteria, primarily due to the presence of valvulopathy (n = 9), elevated aCL IgG antibody levels (n = 6), and/or immunosuppression (n = 3). Of these, 10 received prolonged antibiotic prophylaxis (Figure 2). Three patients received prophylaxis because they were deemed to be at high risk per the treating physician, despite the absence of risk factors defined in this study (Supplementary Table 4). Generally, patients diagnosed with valvopathy were prescribed 12 months of prophylaxis. An increase in phase I IgG to ≥1:512 often prompted further investigations to exclude progression to focal persistent disease. Prophylaxis was not extended in patients who had serologic progression (phase I IgG ≥1:512) in the absence of symptoms or radiographic evidence of focal disease. For patients whose sole risk factor for progression was an elevated aCL IgG, prophylaxis was continued until their titers returned to normal levels (≤15 GPL). The most frequently adopted prophylactic regimen was the combination of doxycycline with hydroxychloroquine (n = 9), followed by doxycycline monotherapy (n = 3) and doxycycline with rifampin (n = 1). The median duration of prophylaxis was 333 days (IQR, 168–414; Supplementary Table 4).

**Figure 3.**
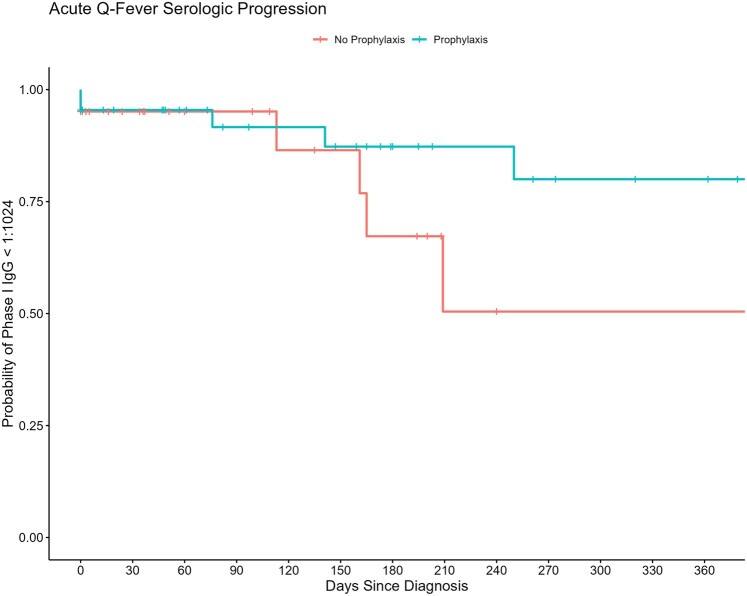
Kaplan-Meier curve of serologic progression in patients with acute Q fever stratified by antibiotic prophylaxis. IgG, immunoglobulin G.

Excluding 5 patients (16.1%) who were lost to follow-up after their initial diagnosis, the median duration of patient monitoring was 206 days (IQR, 63–517). These patients had a median 2 additional Q fever serologies beyond the initial diagnostic test (IQR, 2–4). At the time of the last assessment, 22 patients (71%) had clinical resolution of the infection while 4 (13%) progressed to focal persistent disease, with all developing native valve Q fever infective endocarditis (Table 4). Among those who progressed, 2 had elevated aCL IgG antibody titers as the only identifiable risk factor. The remaining 2 patients did not have aCL IgG antibody levels checked and had no other conventional risk factors for disease progression identified. At 1-year follow-up, 11 patients (30%) demonstrated serologic progression to chronic Q fever (phase I IgG ≥1:1024), 7 of whom did not have clinical evidence of focal persistent disease. Serologic progression in these 7 patients occurred after a median 179 days (IQR, 63–358) following initial diagnosis (Table 4). Prophylactic antibiotic administration was associated with decreased probability of serologic progression in our cohort (Figure 3).

## DISCUSSION

We identified 31 patients with acute Q fever over 10 years at our institution. The majority of the cases were diagnosed in the summer season and were identified in patients residing in the Midwest and Arizona, respectively, likely representing the catchment area of our institution. Over the 10-year period of our cohort study, 4 patients (13%) progressed to focal persistent disease, specifically manifesting as native valve infective endocarditis. The decision to administer antibiotic prophylaxis for preventing progression to persistent disease varied significantly and was largely dependent on the treating physician's judgment. A combination of doxycycline and hydroxychloroquine was most commonly used for prophylaxis. Notably, elevated aCL IgG levels were the only identified risk factor for progression to Q fever endocarditis in 2 patients, despite both having negative TTEs. Due to the limited sample size, no conclusions could be drawn regarding the impact of antibiotic prophylaxis on reducing the risk of disease progression despite an apparent reduction in the probability of serologic progression.

Some of our findings are consistent with prior reports in the literature describing patients with acute Q fever infection. Namely, the male:female ratio for cases reported between 2012 and 2022 was 5.2:1, with the majority having an identifiable exposure history to animal products. While differential occupational exposures to *C burnetii* could explain the higher prevalence of infection in men, some evidence suggests that sex hormones play a role in the predisposition of men to this pathogen [10, 23]. Moreover, most cases were diagnosed during summer. This may be related to changes in patterns of cattle husbandry, grazing, and birthing and to increased exposure of patients to *C burnetii* during the farming season [7, 10, 24]. Consistent with published reports [6, 10, 11, 25, 26], acute hepatitis, flu-like illness, and pneumonia were the most common clinical syndromes in our cohort. These presentations, however, are nonspecific and could be misleading, potentially resulting in underdiagnosis and significant underestimation of the incidence of acute Q fever in hospitalized patients. Clinicians therefore should consider *C burnetii* infection in these clinical settings, particularly for patients with exposure risk factors (Table 2), regardless of residing in regions with a higher prevalence of reported cases [5–7]. Clinicians should also be aware of the temporal delay between the onset of acute Q fever symptoms and seroconversion, the possible coinfection with *Borrelia* spp or *Anaplasma* spp in endemic areas, the serologic cross-reactivity or coinfection with *Bartonella* spp [27, 28] or *E chaffeensis* [29, 30], and the low sensitivity of plasma *C burnetii* PCR during this window period [31]. Microbial cell-free DNA next-generation sequencing has emerged as a promising tool that could enable the diagnosis of acute cases prior to seroconversion even when the PCR test result is negative [27]. Yet, this test is costly and not widely accessible, and it requires further evaluation before it can reasonably be integrated into routine clinical practice for the evaluation of fever of unknown origin and atypical syndromes, such as those caused by acute *C burnetii* infection [27].

In our cohort, 7 patients (22.5%) exhibited rare clinical presentations of acute Q fever, similar to cases documented in existing literature. These included 1 case of acute membranoproliferative glomerulonephritis without infective endocarditis [32, 33], 2 cases of lymphadenitis [11, 34], 1 case of acute pancreatitis [26, 35], 1 case of meningoencephalitis [10, 11], 1 case of hemophagocytic lymphohistiocytosis [11], and 1 case of acute Q fever endocarditis [11, 16, 34, 36]. Elevated aCL IgG (>22 GPL) in the acute phase has been identified as an independent risk factor for many of these complications [11, 34, 36, 37]. It is probable that such presentations are not inherently rare but rather are underrecognized as being secondary to acute Q fever.

With the growing use of PET-CT imaging, lymphadenitis is increasingly being recognized as a manifestation of acute and persistent Q fever infection. It is now identified as a prelymphomatous stage, especially in cases of persistent disease [11, 38, 39], although progression to lymphoma has been noted in patients with acute Q fever lymphadenitis, albeit less frequently [38]. While this observation may not justify PET-CT scans for all patients with acute Q fever, high aCL IgG and palpable superficial lymphadenopathy or identification of deep lymphadenopathy on computed tomography imaging should prompt further investigations, as well as close clinical and serologic monitoring.

Melenotte et al recently introduced criteria for diagnosing cases of possible and definite acute Q fever endocarditis [34]. Recognizing and treating these patients is important, as current evidence suggests that they are at increased risk (up to 6-fold) for progression into persistent endocarditis [10, 11, 34, 36]. In our cohort, only 1 patient was diagnosed with definite acute Q fever endocarditis. However, this condition may have been underdiagnosed, as aCL IgG antibody levels were measured in only 8 patients (26%), which may have resulted in fewer TEEs being performed, especially in cases where TTE findings were negative or inconclusive [34].

Treatment of patients with symptomatic acute Q fever infection, ideally initiated within the first 3 days of symptom onset, has been shown to reduce the duration of illness, risk for hospitalization, and severe complications [3, 10, 40]. In patients who are intolerant to doxycycline, acceptable alternatives include fluoroquinolones, minocycline, clarithromycin, and trimethoprim-sulfamethoxazole [3, 41, 42]. Yet, the regimen, duration, and clinical impact of antibiotic prophylaxis for patients with risk factors for disease progression are less well established.

First and foremost, there is no consensus on defining “high risk” factors, leading to considerable variability across studies. Risk stratification often involves a combination of host factors and laboratory parameters, including aCL IgG positivity (Table 1). Second, the ideal prophylactic regimen and duration remain undefined [43], and the efficacy, safety, tolerability, and risk-benefit ratio of any suggested prophylaxis have yet to be evaluated in a randomized clinical trial. In a study by Million et al [16], a 12-month course of doxycycline and hydroxychloroquine was suggested in high-risk patients with acute Q fever to decrease the risk of Q fever endocarditis, noting that doxycycline monotherapy does not offer protection against endocarditis. Meanwhile, CDC guidelines lack specific recommendations for antibiotic prophylaxis in high-risk cases but advocate for regular clinical assessments and serologic monitoring to facilitate early disease progression management [3]. Health care providers are therefore tasked with navigating the existing literature to conduct individualized risk stratification and discuss the risk-benefit of antibiotic prophylaxis with their patients.

Eight patients (26.7%) in our study were administered a combination of doxycycline and hydroxychloroquine at the time of diagnosis, which was continued as prophylaxis due to their categorization by the treating physician as being at increased risk for progression to focal persistent disease, while for 1 patient, hydroxychloroquine was added to doxycycline on day 14. Other prophylactic regimens included doxycycline monotherapy (n = 3) and a combination of doxycycline with rifampin (n = 1). It is worth pointing out that 1 patient with elevated aCL IgG levels (57.1 GPL) progressed to Q fever endocarditis while undergoing doxycycline monotherapy prophylaxis, whereas another patient (89.4 GPL) did not progress after 12 months with the same regimen. Both patients had no abnormalities on TTE. However, neither underwent TEE to evaluate for potential occult valvular changes that might not have been initially detected.

Three patients developed adverse events while receiving antibiotic prophylaxis, requiring interruption of therapy. These included gastrointestinal intolerance in a patient taking rifampin and doxycycline, severe phototoxicity due to doxycycline despite precautionary measures, and retinal toxicity in a patient prescribed hydroxychloroquine. The small sample size and study design preclude drawing definitive conclusions about the efficacy of antibiotic prophylaxis in preventing progression to focal persistent disease.

This study has several limitations. First, due to the retrospective design, the quality and availability of data are limited by the inherent accuracy of medical records. In addition, variables such as exposure history, which rely on self-reporting, are prone to reporting bias. Our team implemented all feasible measures to ensure data accuracy and reduce the risk of information bias. Second, the study is not powered enough to detect treatment effects due to the small sample size. Furthermore, this was a single-center study, and the generalizability of our findings may be limited, as they might not reflect the full spectrum of management and treatment outcomes across the United States. Additionally, the follow-up period was short for some patients, limiting further assessment of disease progression. Finally, our results and conclusions are partly contingent on the high-risk criteria used in this study.

## CONCLUSIONS AND FUTURE DIRECTIONS

The diagnosis of acute Q fever presents significant challenges, compounded by the array of clinical presentations and extensive body of literature, as well as the lack of clear, comprehensive guidelines that govern the management of this disease. This has resulted in a variety of diagnostic and therapeutic approaches in clinical practice, often leaving clinicians with more questions than answers. Funding and conducting randomized clinical trials is crucial to address several key areas:

- Validating and standardizing the factors considered high risk for progression to focal persistent disease
- Standardizing screening protocols for the identification of high-risk patients
- Establishing the optimal antibiotic regimen and duration and determining the subgroup of high-risk patients who may benefit from chemoprophylaxis (this has been emphasized for Q fever endovascular infections [44])
- Development of standardized evidence-based guidance for serologic follow-up tailored to the most common *C burnetii* strains responsible for human disease in the United States, highlighting key factors that can be used by clinicians to differentiate clinical vs serologic progression.

Concentrating on these key points may facilitate the development of updated and comprehensive clinical guidelines that offer more definitive guidance for clinicians. This can lead to reduced variability in clinical practice and enhanced outcomes for patients with acute Q fever infection.

## Supplementary Material

ofae277_Supplementary_Data

## Supplementary Data

Supplementary materials are available at *Open Forum Infectious Diseases* online. Consisting of data provided by the authors to benefit the reader, the posted materials are not copyedited and are the sole responsibility of the authors, so questions or comments should be addressed to the corresponding author.

## Notes

***Patient consent***. The study was performed following approval from the Institutional Review Board at Mayo Clinic Rochester under a waiver of informed consent (22-003559).
