# Supplementary material for: Acute Coxiella burnetii Infection: A 10-Year Clinical Experience at a Tertiary Care Center in the United States
Source: Open Forum Infect Dis. 2024 May 10;11(6):ofae277. doi: 10.1093/ofid/ofae277 (PMC11167673; doi:10.1093/ofid/ofae277)

Table of Contents

[Definitions: 2](#_Toc162531171)

[Additional serological and clinical characteristics of the patients with acute Q fever but Phase I IgG ≥ 1:1024 2](#_Toc162531172)

[Supplementary Tables 2](#_Toc162531173)

[Supplementary Figures 5](#_Toc162531174)

# Definitions:

**Seasons of the year:**

Winter (December to February), Sprint (March to May), Summer (June to August) and Fall (September to November)

**Immunosuppression:**

Immunosuppression was defined as having one or more of the following conditions: solid or hematologic malignancy undergoing active chemotherapy, an HIV infection with a CD4 count ≤200 / mm3, past history of solid organ or bone marrow transplant actively receiving immunosuppressive agents, inherent immunodeficiency including asplenia, or the use of immunosuppressant drugs for the management of autoimmune disorders (3, 10).

# Additional serological and clinical characteristics of the patients with acute Q fever but Phase I IgG ≥ 1:1024

| Patient | Phase I IgG | Phase II IgG | Phase I IgM | Phase II IgM | Clinical presentation | Other |
| --- | --- | --- | --- | --- | --- | --- |
| 1 | 8192 | 16000 | 128 | 32000 | Fever, malaise, acute hepatitis | No progression |
| 2 | 2048 | 16384 | 128 | 256 | Fever, malaise | Evidence of seroconversion and confirmed acute Q fever. Negative TTE, progressed to native valve endocarditis |
| 3 | 1024 | 4096 | 64 | 16 | Fever, malaise, acute hepatitis | No progression |
| 4 | 1024 | 8192 | 1024 | 16 | Fever, malaise, cervical lymphadenopathy | Lymph node biopsy with negative Q fever PCR, and histopathology with necrotizing lymphadenitis. Negative TTE. No progression |

# Supplementary Tables

| Supplementary Table 1: Q fever classification |  |
| --- | --- |
| **Acute Q fever confirmatory laboratory criteria – n (%)** | 9 (29.0) |
| 4- fold rise in phase II IgG between paired sera | 8 (25.8) |
| *Coxiella burnetii* PCR in tissue | 1 (3.2) |
| *Coxiella burnetii* PCR positive in blood | 0 (0) |
| *Coxiella burnetii* in tissue by immunohistochemistry | 0(0) |
| *Coxiella burnetii* isolated by culture | 0(0) |
| **Acute Q fever supportive criteria -n (%)** | 22 (88.0) |
| Single IgG phase II >=128 by IFA | 22 (71.0) |
| Elevated phase II IgG or IgM by ELISA or Latex agglutination | 0(0) |
| **CDC case classification of acute Q fever *** |  |
| Confirmed acute Q fever | 9 (29.0) |
| Probable acute Q fever | 22 (71.0) |

* **Confirmed acute Q fever**: Laboratory-confirmation with clinical evidence of infection or an epidemiological link to a laboratory-confirmed case; **Probable acute Q fever**: clinical evidence of infection with laboratory-supportive results(1)

| Supplementary Table 2: Additional Serologies obtained during the initial workup |  |
| --- | --- |
| Bartonella serology obtained - n (%) | 17 (54.8) |
| Serology positive | 3 (17.6) |
| Tick-borne disease antibody panel obtained – n (%) | 19 (61.3) |
| Antibody panel positive | 4 (21.1) |
| Anaplasma | 3 (15.8) |
| Ehrlichia | 1 (5.3) |
| Lyme | 1 (5.3) |
| Babesia | 0 (0) |

| Supplementary Table 3: Abnormal imaging findings |  |  |
| --- | --- | --- |
| Abnormal TTE – n (%) * | 5 / 17 (29.4) |  |
| Valvulopathy ^β^ | 4 (23.5) |  |
| Prosthetic valve | 1(5.9) |  |
| Vegetation | 1 (5.9) |  |
| Degenerative strands | 0(0) |  |
| Ruptured chordae associated with valvular regurgitation | 1(5.9) |  |
| Abnormal TEE – n (%) | 6 / 11 (54.5) |  |
| Valvulopathy | 5 (45.5) | |
| Prosthetic valve | 0(0) | |
| Vegetation | 1 (9.1) | |
| Degenerative strands | 2 (18.2) | |
| Other | 2 (18.2) | |
| Myxomatous mitral valve leaflets. At least two major ruptured chordae to flail segment of posterior mitral leaflet. | 1 / 2 |  |
| Tricuspid aortic valve with a small mobile filamentous lesion on the non−coronary cusp. The appearance suggested a Lamb’s excrescence rather than vegetation | 1 / 2 |  |
| Abnormal PET- CT – n (%) | 6 / 10 (60.0%) |  |
| Presence of vascular aneurysm without FDG uptake | 0 (0) |  |
| Presence of vascular graft without FDG uptake | 0 (0) |  |
| Presence of vascular aneurysm with FDG uptake | 0 (0) |  |
| Presence of vascular graft with FDG uptake | 0 (0) |  |
| Other | 6 (100) |  |
| Bilateral upper lung infiltrates/consolidation | 1 (16.7) |  |
| Increased uptake in the liver | 1 (16.7) |  |
| Lymphadenopathy # | 2 (33.3) |  |
| Mild FDG uptake along the posterior margin of the aortic valve prosthesis^ | 1 (16.7) |  |
| Splenomegaly | 1 (16.7) |  |

* 17 patients had a TTE , of which, 3 had a follow-up TEE

β Valvulopathy defined as presence of one or more of the following: bicuspid aortic valve, grade 2 or more valvular stenosis, Grade 2 or more valvular regurgitation, mitral valves prolapse, valve thickening, destruction, or remodeling, vascular aneurysm

# Lymph node biopsy performed in one patient with diffuse lymphadenopathy and was negative for malignancy

^ Patient with bacterial prosthetic aortic valve infective endocarditis and acute Q fever infection requiring aortic valve replacement. Q fever polymerase chain reaction obtained on two separate aortic valve specimens returned negative. Q fever phase I IgG consistently < 1:1024

| Supplementary Table 4: Detailed Rationale for Prolonged Antibiotic Prophylaxis to Prevent Progression to Focal Persistent Disease Irrespective of Presence of Conventional Risk Factors and Selection of Antibiotic Regimens | Number of patients  (Total = 13) |
| --- | --- |
| Concerns regarding a vegetation seen on echocardiography, although it is highly probable that the underlying cause was Lambl's excrescence * | 1 |
| Upward trend in Phase I IgG, and transthoracic echocardiogram (TTE) showed trivial aortic regurgitation * | 1 |
| Very high Phase II IgG and Phase II IgM levels * | 1 |
| Concomitant diagnosis with bacterial infective endocarditis necessitating valve replacement and acute Q fever | 1 |
| High anticardiolipin IgG antibody levels alone | 3 |
| High anticardiolipin IgG antibody levels associated with an upward trend in Phase I IgG. | 1 |
| High anticardiolipin IgG antibody levels and aortic valve sclerosis | 1 |
| Immunocompromised state | 2 |
| Acute Q fever endocarditis as evidenced by Phase II IgG 1024, phase II IgM 64 and evidence of chordae tendineae rupture on TTE β | 1 |
| Fluctuating Phase I IgG titers and presence of mitral valve prolapse. | 1 |
| **Antibiotics used for prophylaxis** |  |
| Doxycycline | 3 (23) |
| Doxycycline + hydroxychloroquine | 9 (69) |
| Doxycycline plus rifampin | 1 (8) |
| Duration of prophylaxis, days – Median (IQR) | 333.0 (168.0, 414.0) |

* Deemed by the treating physician as high-risk features warranting prophylaxis despite absence of conventional risk factors

Β Following criteria outlined by Melenotte C et al. (2)

References

1. Centers for Disease Control and Prevention (CDC). Diagnosis and Management of Q Fever — United States, 2013: Recommendations from CDC and the Q Fever Working Group. MMWR Recommendations and reports: Morbidity and mortality weekly report 2013;62(RR-03):1-30.

2. Eldin C, Mélenotte C, Mediannikov O, Ghigo E, Million M, Edouard S, et al. From Q Fever to Coxiella burnetii Infection: a Paradigm Change. Clin Microbiol Rev. 2017;30(1):115-90.

# Supplementary Figures

Figure S1: Study flow chart.


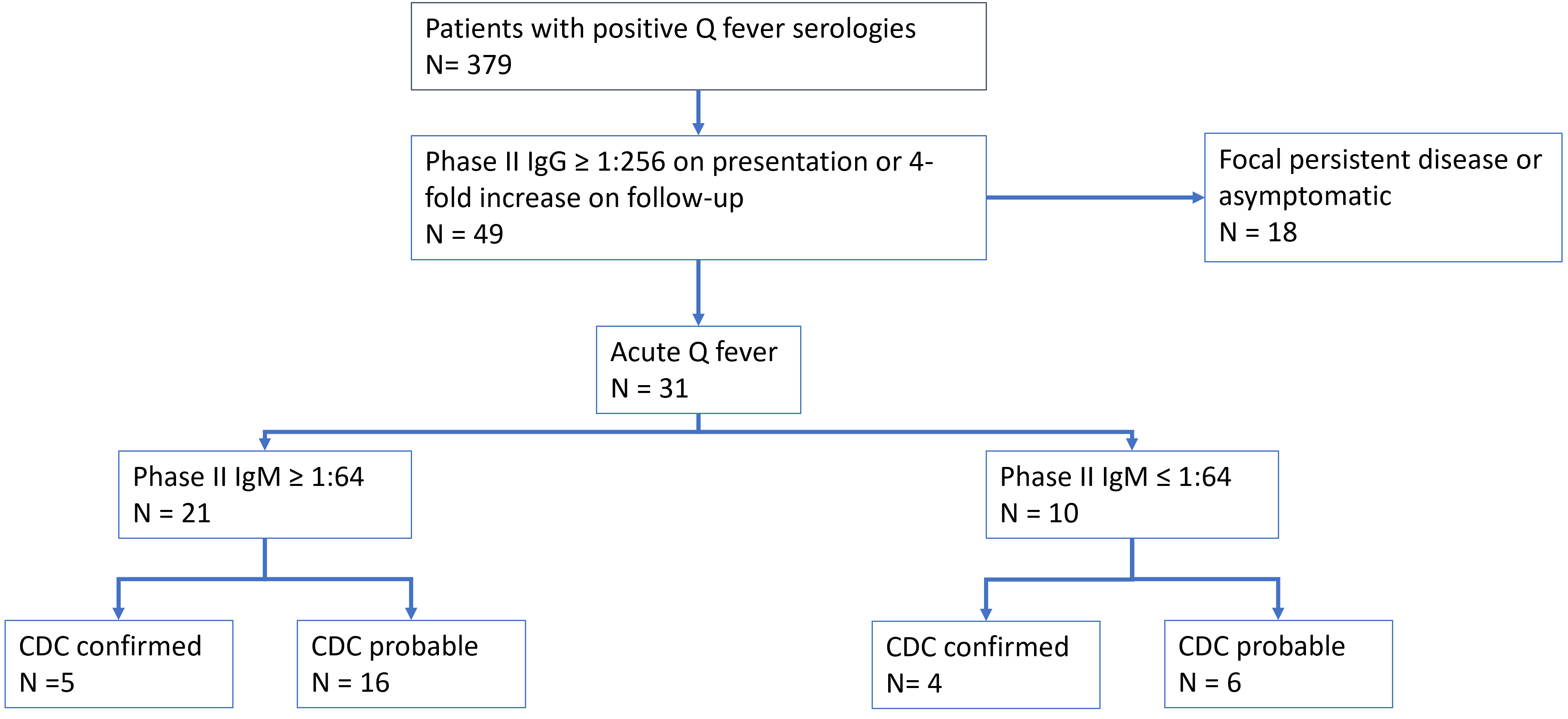


Figure S2: Average Number of Patients with Acute Q fever Diagnosed Per Year


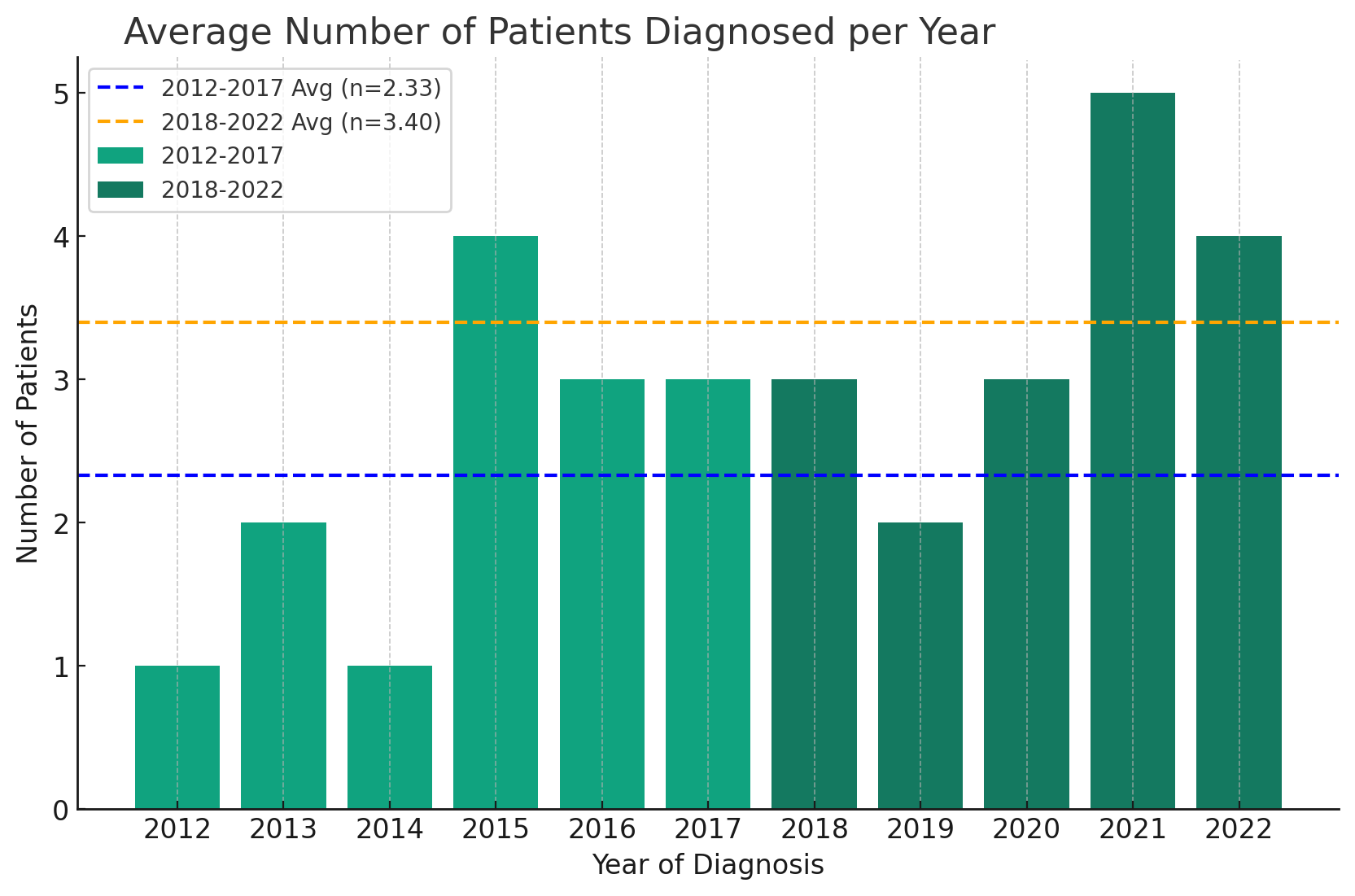

Supplement: ofae277_Supplementary_Data [file ofae277_supplementary_data.docx]
